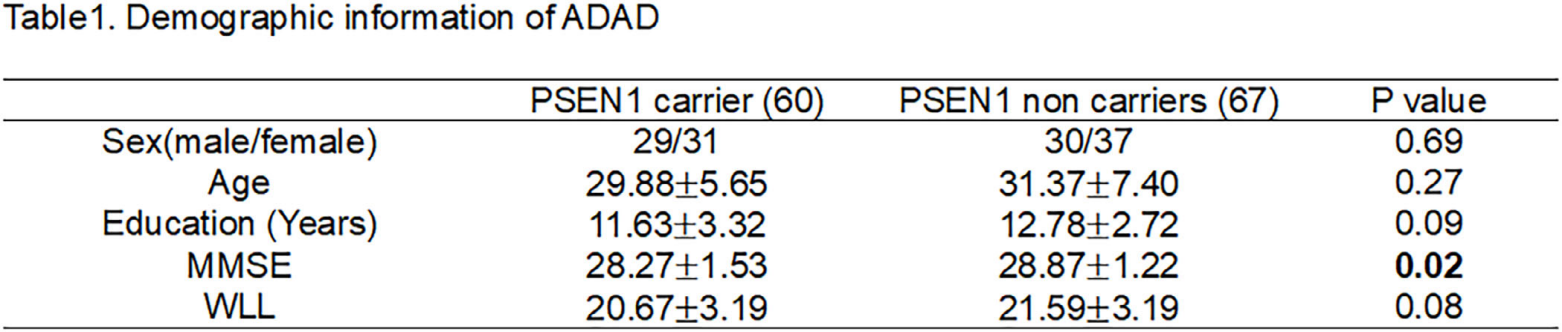# Early changes in basal forebrain volume and cognitive function in preclinical autosomal dominant Alzheimer's disease

**DOI:** 10.1002/alz70856_105027

**Published:** 2026-01-07

**Authors:** Bing He, Paula Ospina, Alejandro Espinosa, Juan Camilo Becerra‐Mateus, Laura Osorio, Diana Alzate, Sergio Alvarez, Alice Grazia, Stefan Teipel, Vincent Malotaux, Catarina Tristão‐Pereira, Meredith Rowe, Averi Giudicessi, Sonia Do Carmo, David Fernando Aguillón Niño, A. Claudio Cuello, Yakeel T. Quiroz

**Affiliations:** ^1^ Massachusetts General Hospital, Harvard Medical School, Boston, MA, USA; ^2^ Grupo de Neurociencias de Antioquia, University of Antioquia, Colombia, Medellín, Antioquia, Colombia; ^3^ Grupo de Neurociencias de Antioquia, Facultad de Medicina, Universidad de Antioquia, Medellín, Antioquia, Colombia; ^4^ Hospital Pablo Tobón Uribe, Medellín, Antioquia, Colombia; ^5^ Genman Center for Neurodegenerative Disease‐Rostock/Greifswald, Rostock, German, Rostock, Germany; ^6^ Department of Psychosomatic Medicine, University Medicine Rostock, Rostock, Germany, Rostock, Germany; ^7^ Boston University, Boston, MA, USA; ^8^ McGill University, Montreal, QC, Canada

## Abstract

**Background:**

The basal forebrain (BF), home to cholinergic neurons essential for attention and memory undergoes structural changes in sporadic Alzheimer's Disease and in at‐risk individuals, contributing to cognitive decline. To investigate whether BF volume is reduced in preclinical autosomal‐dominant Alzheimer's disease (ADAD), we studied cognitively‐unimpaired carriers of the PSEN1 E280A mutation from the Colombian kindred, the largest ADAD cohort with a single mutation, known for early cognitive decline (mild cognitive impairment at age 44, dementia at 49). Age was used as a proxy for disease progression to analyze BF volume and its relationship with age and cognitive performance.

**Method:**

This study included 127 cognitively‐unimpaired individuals from the PSEN1 Colombian kindred (60 carriers, 67 non‐carriers; mean‐age: 30.67 ± 6.65 years; mean‐education: 12.24 ± 3.06 years). Unimpaired status was defined by Functional Assessment Staging (FAST) scores <2. Participants underwent structural MRI and cognitive testing, with BF volumes measured using a cholinergic nuclei map. Cognition was assessed with the Mini‐Mental State Examination (MMSE) and the CERAD Word List Learning (WLL) task. BF volume differences between groups were assessed using a t‐test, and partial Pearson correlations (adjusted for sex, education, and intracranial volume) were used to evaluate relationships between BF volume, age, and cognition.

**Result:**

BF volumes did not differ significantly between carriers (693.83 ± 68.3 mm3) and non‐carriers (694.00 ± 65.24 mm3) (*p* =  0.82). Age was negatively correlated with BF volume in the overall sample (*r* = ‐0.41, *p* =  2.7e‐06), carriers (*r* = ‐0.41, *p* =  0.001), and non‐carriers (*r* = ‐0.44, *p* =  2.6e‐04). However, BF volume showed no significant correlation with MMSE or WLL in the overall sample, carriers, or non‐carriers.

**Conclusion:**

These findings suggest that Alzheimer's‐related volumetric changes in the basal forebrain may not manifest during the early preclinical stages of ADAD. This highlights the importance of future studies incorporating longitudinal measures to track BF changes over time, spanning the continuum from preclinical to clinical stages. Such research could provide critical insights into the temporal dynamics of BF involvement and its potential as a marker for early detection or therapeutic target in ADAD.